# Detection of AZF microdeletions and reproductive hormonal profile analysis of infertile sudanese men pursuing assisted reproductive approaches

**DOI:** 10.1186/s12894-021-00834-3

**Published:** 2021-04-23

**Authors:** Hassan Osman Alhassan Elsaid, Tarteel Gadkareim, Tagwa Abobakr, Eiman Mubarak, Mehad A. Abdelrhem, Dalya Abu, Elsir Abu Alhassan, Hind Abushama

**Affiliations:** 1 Department of Zoology, Faculty of Science, University of Khartoum, P.O. Box 321, 11115 Khartoum, Sudan; 2 Elsir Abu Alhassan Fertility Centre, Khartoum, Sudan

**Keywords:** Male infertility, Azoospermia, Oligozoospermia, Y chromosome microdeletion, AZF, Reproductive hormones

## Abstract

**Background:**

Male factor is the major contributor in roughly half of infertility cases. Genetic factors account for 10–15% of male infertility. Microdeletions of azoospermia factors (AZF) on the Yq region are the second most frequent spermatogenesis disorder among infertile men after Klinefelter syndrome. We detected in our previous study a frequency of 37.5% AZF microdeletions which investigated mainly the AZFb and AZFc. We attempted in this study for the first time to evaluate the frequencies of all AZF sub-regions microdeletions and to analyze reproductive hormonal profiles in idiopathic cases of azoospermic and oligozoospermic men from Sudan.

**Methods:**

A group of 51 medically fit infertile men were subjected to semen analysis. Four couples have participated in this study as a control group. Semen analysis was performed according to WHO criteria by professionals at Elsir Abu-Elhassan Fertility Centre where samples have been collected. We detected 12 STSs markers of Y chromosome AZF microdeletions using a multiplex polymerase chain reaction. Analysis of reproductive hormone levels including Follicle Stimulating, Luteinizing, and Prolactin hormones was performed using ELISA. Comparisons between outcome groups were performed using Student’s t-test Chi-square test or Fisher’s exact test.

**Results:**

AZF microdeletion was identified in 16 out of 25 Azoospermic and 14 out of 26 of the Oligozoospermic. Microdeletion in the AZFa region was the most frequent among the 30 patients (N = 11) followed by AZFc, AZFd (N = 4 for each) and AZFb (N = 3). Among the Oligozoospermic participants, the most frequent deletions detected were in the AZFa region (N = 10 out of 14) and was significantly associated with Oligozoospermic phenotype, Fisher's Exact Test (2-sided) *p* = 0.009. Among the Azoospermic patients, the deletion of the AZFc region was the most frequent (N = 9 out of 16) and was significantly associated with Azoospermia phenotype Fisher's Exact Test *p* = 0.026. There was a significant difference in Y chromosome microdeletion frequency between the two groups. The hormonal analysis showed that the mean levels of PRL, LH, and FSH in Azoospermic patients were slightly higher than those in oligozoospermic. A weak negative correlation between prolactin higher level and Azoospermic patients was detected. (AZFa r = 0.665 and 0.602, *p* = 0.000 and 0.0004, AZFb r = 0.636 and 0.409, *p* = 0.000 and 0.025, and AZFd r = 0.398 and 0.442, *p* = 0.029 and 0.015). The correlation was positive for AZFa and negative for AZFb and AZFd.

**Conclusions:**

We concluded in this study that the incidences of microdeletions of the Y chromosome confined to AZF a, b, c and d regions is 58.8% in infertile subjects with 31.4% were Azoospermic and 27.5% were Oligozoospermic. This might provide a piece of evidence that these specified regions of the Y chromosome are essential for controlling spermatogenesis. These findings will be useful for genetic counseling within infertility clinics in Sudan and to adopt appropriate methods for assisted reproduction.

## Background

Infertility is a common medical problem affecting 15% of those who are trying to conceive a pregnancy. In 30% to 50% of the cases, a male is the major contributor to the problem [1]. Amongst many contributing factors for infertility, genetic factors alone constitute 10–15% [2]. Chromosomal anomalies whether numerical or structural was placed as the first of these factors as in the case of Klinefelter syndrome [3]. Y-chromosome microdeletions with a frequency of 1% to 50% are considered the second most frequent genetic causes that result in azoospermia and are directly associated with male infertility [4].

In the Y chromosome, the azoospermia factor (AZF) region is irreplaceable in spermatogenesis. A previous study has shown that Microdeletions in the AZF region will lead to spermatogenic failure and therefore testicular sperm extraction is not recommended for the patient that carries these microdeletions [3]. Y chromosome microdeletions are more common in infertile males than in the general population and previous studies revealed that these microdeletions are found in 3–5% of Oligozoospermic patients and 6–16% Azoospermic patients [3].

An infertility rate of 11.5% has been reported previously in 10 out of 18 Sudanese states. In Khartoum, there are around 12 private well-established fertility centers that providing assisted reproductive technology services but none of them has carried out surveys to explore the genetic causes of infertility. A Recent study has recorded an increase in the high rate of primary infertility in Khartoum [5]. We detected in our previous study a frequency of 37.5% AZF microdeletions which investigated mainly the AZFb and AZFc patients [6]. The study was performed on thirty-two males who were referred to Elsir Abu-Elhassan fertility clinic following the same procedures of semen analyses and DNA markers that have been used in the present study. The highest rate of microdeletion was found at the AZFc subregion (50%) followed by 33.3% at AZFb loci and 16.7% was found to be at both loci AZFb + c. We are intending in this study to assess the occurrence of different types of Y chromosome microdeletions in a group of infertile Sudanese men and to analyze their hormonal profile. Furthermore, we will compare our results with the reported regional result in the Afro-Arab region and the internationally reported results.

## Methods

### Study participants

The study was conducted at Elsir Abu-Elhassan fertility center in the capital of Sudan, Khartoum. The center receives its clients from all over the country. A group of 51 male subjects from those enrolled at the center was chosen for this study. All study participants were primarily infertile. Participants were refined based on the following criteria: had failed to conceive or produce progeny after one year of unprotected sexual intercourse, medically fit, free of any congenital disorders, diagnosed with non-obstructive infertility, enjoying normal sexual life [5, 6]. All participants underwent physical examination, semen analysis, reproductive hormone estimation, and Y chromosome microdeletion analyses.

A group of 4 couples has participated in this study as a control, was not diagnosed with any congenital problem, and they were successfully parents during the first year of their unprotected intercourse. The control group was used to assure the diagnostics experiment. Sudanese are a very conservative community and we faced difficulties to involve more voluntary control groups in such studies.

### Ethical statement

Before commencement, ethical clearance was granted from the scientific research administration, Federal Ministry of Health, Khartoum, Sudan. Written consent was obtained from each participant.

### Semen analysis

Semen analysis was performed twice with a standard abstinence period between each sample to confirm infertility according to WHO criteria [5] by professionals at Dr. Elsir Abu-Elhassan Fertility Centre. Briefly, semen ejaculate from each study participant was obtained by masturbation at the fertility center after at least three days of abstinence. 10 µL of each semen sample was then examined for the number of sperms under a phase-contrast microscope using a special counting chamber. Those with a sperm concentration of less than 15 × 10^6^ sperm/mL were denoted as Oligozoospermic while those with no sperms in their ejaculates were denoted as Azoospermic [5]. The absence of sperms was confirmed even further by using centrifugation for each sample at 12,000 rpm for fifteen minutes.

### Y-Chromosome microdeletion

Microdeletions in the Y chromosome were investigated using multiplex polymerase chain reaction (m-PCR). Briefly, Blood samples were collected in an EDTA vacutainer tube. Genomic DNA was extracted from the blood using a modified salting-out method. Extracted DNA was -quantitatively and qualitatively- assessed on the NANO DROP spectrophotometer (ND-1000).

Microdeletions were detected in 4 conventional Azoospermia factors loci at the q arm of the Y chromosome, AZFa, AZFb, AZFc, and AZFd. AZFb and AZFc, are usually overlapping. Similarly, the proximal portion of AZFc is called AZFd, but the usefulness of isolating this subregion remains unclear [7]. The sex-determining region (SRY) at the Yp arm was used as an internal positive control in the Y chromosome. Sequence-tagged sites STSs in AZFa, and c was covered using 3 pairs of primers for each, while 2 pairs were used for AZFb and AZFd. One pair of primers was used for SRY as illustrated in Table 1.

**Table 1:** Sequence-tagged sites and primer sequences for Y chromosome microdeletion analysis

Region	STS		Sequence 5′– 3'	Size bp	References
Yp	sY14	Forward	GAATATTCCCGCTCTCCGGA	495	[9]
		Reverse	GCTGGTGCTCCATTCTTGAG		
AZFa	sY81	Forward	AGGCACTGGTCAGAATGAAG	209	[8]
		Reverse	AATGGAAAATACAGCTCCCC		
	sY84	Forward	AGAAGGGTCTGAAAGCAGGT	326	[9]
		Reverse	GCCTACTACCTGGAGGCTTC		
	sY86	Forward	GTGACACACAGACTATGCTTC	320	[9]
		Reverse	ACACACAGAGGGACAACCCT		
AZFb	sY127	Forward	GGCTCACAAACGAAAAGAAA	274	[9]
		Reverse	CTGCAGGCAGTAATAAGGGA		
	sY128	Forward	GGATGAGACATTTTTGTGGG	384	[10]
		Reverse	AGCCCAATGTAAACTGGACA		
AZFc	sY239	Forward	CATTCATCTTCCCTTTTGAAGG	201	[8]
		Reverse	ATGCAAGTCGCAGGAAATCT		
	sY254	Forward	GGGTGTTACCAGAAGGCAAA	370	[9]
		Reverse	GAACCGTATCTACCAAAGCAGC		
	sY255	Forward	GTTACAGGATTCGGCGTGAT3	126	[9]
		Reverse	CTCGTCATGTGCAGCCAC		
AZFd	sY152	Forward	AAGACAGTCTGCCATGTTTCA	125	[11]
		Reverse	ACAGGAGGGTACTTAGCAGT		
	sY153	Forward	GCATCCTCATTTTATGTCCA	139	[8]
		Reverse	CAACCCAAAAGCACTGAGTA3		

Three m-PCRs were applied for each participant. The PCR profile was performed following [8]. All PCR products were allowed to run in 1X TBE dissolved 2% agarose gel. Electrophoresis was performed in 1X TBE buffer at a voltage of 86 V for 45 min. The documentation performed using the WISD documentation system. Each sample was scored using a 100 base pair DNA ladder. Each experiment was performed at least twice.

### Hormonal analysis

ELISA assay was performed to assess the level of three reproductive hormones in the study group. Briefly, collected blood samples were centrifuged at 3000 g for 20 min at room temperature to extract sera. Serum from each participant was then placed into a plain tube and kept frozen at -20 °C until analyzed.

The analyzed hormones were Follicle Stimulating hormone (FSH), Luteinizing Hormone (LH), and Prolactin (PRL). PRL (Cat.NO 53030), LH (Cat.NO. 53010), and FSH (Cat. No. 53020). ELISA assay kit from Human Biochemica und Diagnostica GmbH was used to measure serum reproductive hormones using multiplex enzyme-linked immunosorbent assay (ELISA) according to manufacturer instruction.

### Statistical analysis

Data were expressed as the mean ± the standard deviation (SD) or number (percentage %). Comparisons between outcome groups were performed using Student’s t-test for continuous variables and Chi-square test or Fisher’s exact test for categorical variables. Statistical significance was considered when *p* ≤ 0.05. Statistical analysis was carried out using the Statistical Package for Social Sciences version 25 (SPSS Inc., Chicago, IL, USA).

## Results

**Fig. 1:**
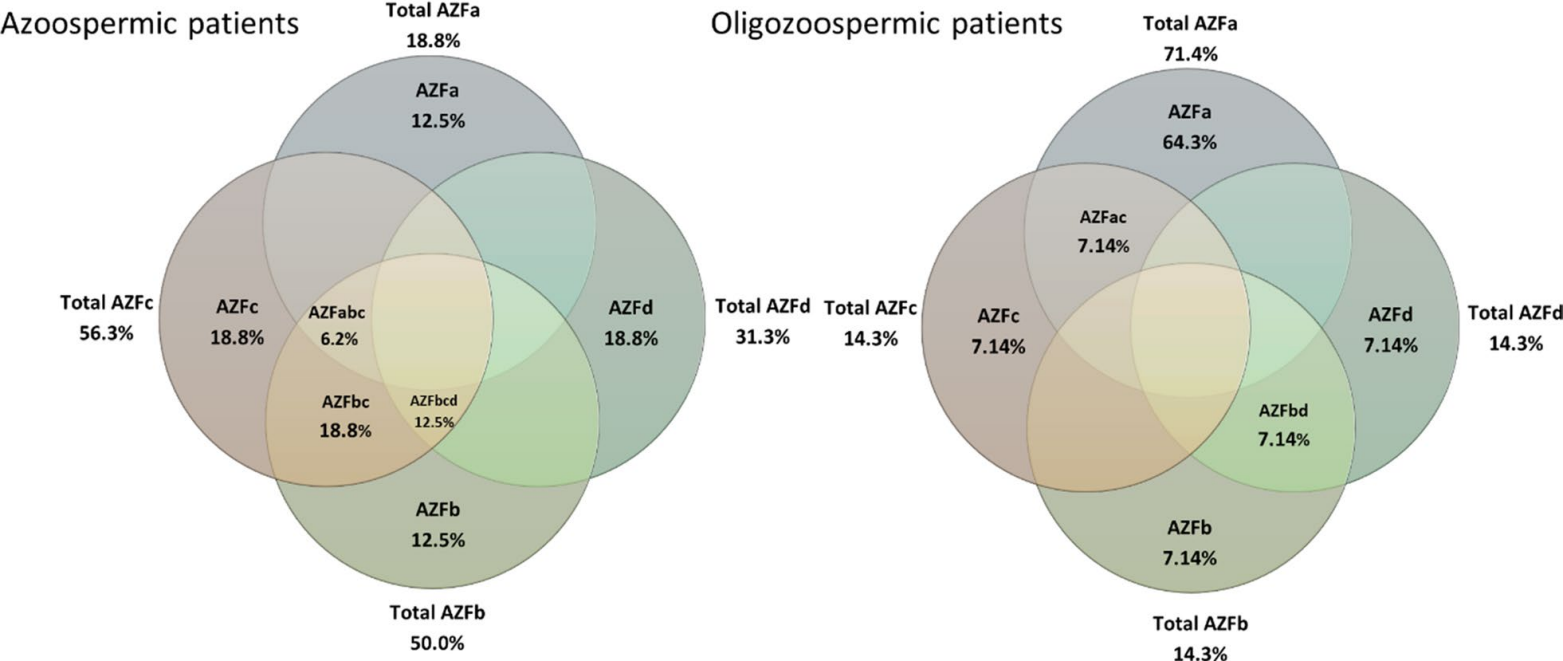
Diagram manifests the percentage of AZF microdeletion in Azoospermic and Oligozoospermic patients. Total AZFa, AZFb, AZFc, and AZFd show the percentage of the deletion including the combination

This study was performed on 51 infertile Sudanese men, medically healthy at the time of this study, with no chromosomal anomaly and diagnosed with non-obstructive Azoospermia or Oligozoospermia. According to the semen analysis results, participants were grouped into two, Azoospermic (no sperms detected) and Oligozoospermic (low sperm concentration than normal 15 × 10^6^ sperm/mL). None of the control participants was shown any of the studied microdeletions. The overall frequency of Y chromosome microdeletions was 58.8% (30/51), 31.4% were Azoospermic and 27.5% were Oligozoospermic. Among the two categories of the semen analyses, 64% (N = 25) of the Azoospermic and 53.8% of the Oligozoospermic were having at least one of the AZF subgroup microdeletions as shown in Table 2. Of the 30 patients with Y chromosome microdeletions in both the Azoospermic and Oligozoospermic, the deletion of the AZFa region was the most frequent (36.7%, N = 11) followed by AZFc and AZFd (13.3% each, N = 4) and AZFb (10%, N = 3). The most frequent combination was AZFbc with the same frequency of AZFb 10% (N = 3), followed by AZFbcd 6.7% (N = 2) while AZFac, AZFabc, and AZFbd sharing 3.3% N = 1) for each as illustrated in Fig. 1.

**Table 2:** The frequencies and types of Y chromosome microdeletions in infertile participants with azoospermia or Oligozoospermia

	Azoospermia (No. 25)	Oligozoospermia (No. 26)	Total N	p value
Microdeletion occurrence	64% (16)	53% (14)	30	
AZFa	12.5% (2 out of 16)	64.3% (9 out of 14)	11	0.009*
AZFb	12.5% (2 out of 16)	7.1% (1 out of 14)	3	> .05
AZFc	18.8% (3 out of 16)	7.1% (1 out of 14)	4	0.026*
AZFd	18.8% (3 out of 16)	7.1% (1 out of 14)	4	> .05
AZFbc	18.8% (3 out of 16)	0% (0 out of 14)	3	> .05
AZFbcd	12.5% (2 out of 16)	0% (0 out of 14)	2	> .05
AZFabc	6.3% (1 out of 16)	0% (0 out of 14)	1	> .05
AZFbd	0% (0 out of 16)	7.1% (1 out of 14)	1	> .05
AZFac	0% (0 out of 16)	7.1% (1 out of 14)	1	> .05

* very significant

Among the Azoospermic patients, the deletion of the AZFc region was the most frequent (56.3%) and was significantly associated with Azoospermia phenotype Fisher's Exact Test *p* = 0.026. However, the results of multiple combination deletions have shown that AZFa, AZFb, AZFbcd microdeletions were sharing the same frequency of 12.5%, while AZFc, AZFd, and AZFbc detected with a frequency of 18.8% and AZFabc was the lowest frequent deletion with a frequency of 6.3%. AZFbd and AZFac deletion combination were not detected in the Azoospermic participants as illustrated in Table 2.

Among the Oligozoospermic participants, the most frequent deletions detected were in the AZFa region with a total frequency of 71.4% and was significantly associated with Oligozoospermic phenotype, Fisher's Exact Test (2-sided) *p* = 0.009. All other reported microdeletions (AZFb, AZFc, AZFd, AZFbd, and AZFac) were at a frequency of 7.1%. No AZFbc, AZFbcd, AZFabc deletion combination was detected in this group of participants.

We found a significant difference in Y chromosome AZF microdeletion frequency between the two groups of semen analysis as illustrated in Table 2.

Serum levels of PRL, LH, and FSH were compared between (Azoospermic and Oligozoospermic—with or without microdeletion) as illustrated in Table 3. Generally, hormonal analysis has shown that prolactin was slightly higher than the normal level in azoospermia participants with microdeletion, 56.3% of Azoospermic have higher prolactin levels compared to 14.3% in the Oligozoospermic. Among the participants without microdeletion, an equal frequency of 33.3% in both Azoospermic and Oligozoospermic have a higher prolactin level.

**Table 3:** Comparison of the Mean hormonal levels in infertile men Azoospermic and Oligozoospermic with and without chromosome microdeletion

	Total	Azoospermia		Oligozoospermia	
		(−)	(+)	(−)	(+)
No. of participants	51	9	16	12	14
Age (Year)	37.08 ± 6.21	38.67 ± 8.12	37.13 ± 4.51	35.17 ± 5.73	37.64 ± 7.12
PRL (ng/ml)	16.77 ±	16.60 ± 7.99	19.51 ± 10.22	17.65 ± 9.93	12.98 ± 5.27
LH (mIU/ml)	11.61 ±	9.20 ± 11.39	10.41 ± 7.71	17.26 ± 24.54	8.87 ± 6.95
FSH (mIU/ml)	16.87 ±	18.89 ± 20.4	21.28 ± 16.82	11.42 ± 6.14	15.22 ± 17.18

LH level was higher than the normal range in all participants with and without microdeletions. In general, Oligozoospermic without microdeletion was noticed to have the highest level of serum LH. Moreover, the LH level was higher in 53.8% of Azoospermic compared to 50% in Oligozoospermic among participants with microdeletion. Among participants without microdeletion, 37.5% of Azoospermic have a higher LH level compared to 41.7% in Oligozoospermic.

FSH serum levels were also higher than the normal range in all participants with and without microdeletions except for the Oligozoospermic without microdeletion. FSH level was higher in 50% of Azoospermic compared to 42.9% in the Oligozoospermic among participants with microdeletion. Among participants without microdeletion, 22.2% of Azoospermic have a higher FSH level compared to 33.3% in Oligozoospermic. In the Oligozoospermic, only 7.7% showed a low FSH level in the presence or absence of the microdeletion. A weak negative correlation was detected between prolactin higher level and Azoospermic patients r = 0.434, *p* = 0.016.

In the present study, we didn’t observe a significant correlation between the mean values of the three hormones with the presence or absence of the AZF microdeletion. However, a very weak correlation was detected between prolactin and the following AZF regions (AZFa r = 0.665 and 0.602, *p* = 0.000 and 0.0004, AZFb r = 0.636 and 0.409, *p* = 0.000 and 0.025, and AZFd r = 0.398 and 0.442, *p* = 0.029 and 0.015). The correlation was positive for AZFa and negative for AZFb and AZFd.

## Discussion

In the present study, we assessed the prevalence of Y chromosome microdeletions in male infertility among the Sudanese population. Structural rearrangements of the Y chromosome abnormalities played a principal role in male infertility including Y chromosome microdeletions which remained poorly studied and little discussed in the literature, including their prevalence in the Sudanese population. The occurrence of chromosomal abnormalities in infertile men was found to be varied between 2.2% and 19.6% in different populations [12] which was found to be inversely affected the sperm count [13].

We managed in our previous study to analyze the microdeletions of the AZF regions and we detected a frequency of 37.5%. AZFc region was the most frequent followed by AZFb [14].

The frequency of Yq microdeletions observed in both oligozoospermic and azoospermic men is compared with the reports from different parts of the world as shown in Table 4. In our study, 64% of the Azoospermic were noticed to have microdeletion while 53.8% of the Oligozoospermic had microdeletion. We have shown a significant difference in Y chromosome microdeletion frequency between the two categories. Previous studies have illustrated the crucial role of the Y chromosome in the process of spermatogenesis. Deletions of the Y chromosome affect the genes controlling spermatogenesis, leading to a defect in sperm production [15, 16]. Y chromosome microdeletion is the second most frequent genetic cause of infertility after Klinefelter syndrome.

**Table 4:** Studies in Some of the Arabic and Middle Eastern countries with different frequencies

References	Year	Region	Study population	Frequency
[51]	2007	Morocco	127	3.15
[21]	2008	Tunisia	146	6.85%
[52]	2012	Morocco	339	3.83%
[53]	2013	Algeria	80	1.25%
[54]	2014	Jordan	100	8.30%
[19]	2015	Morocco	85	18.83%
[55]	2017	Egypt	210	7.14%
[56]	2017	Iran	81	6.17%
[57]	2018	Saudi Arabia	88	2.27%
[58]	2018	Jordan	142	4.93%
[59]	2018	Qatar	179	1.12%
[32]	2020	Iraq	185	47.80%
This study	2020	Sudan	51	58.82%

In the present study, Y chromosome microdeletions were detected in 30 out of 51 patients (58.8%). Out of these 31.4% were found to be Azoospermic and 27.5% were Oligozoospermic with no chromosomal abnormality identified. The outcome of microdeletions frequency in our study is high in comparison to that found in previous studies by Ghorbel et al. (17.1%) [17] and Fayez et al. (20.4%) [18] or in other Arab populations (Morocco (18.83%) [19], Kuwait (2.6%) [20], Tunisia (2.7% and 6.85%) [21, 22], Saudi Arabia (3.2%) [23] and Egypt (4%) [24].

The frequencies of Y chromosome microdeletions differ from one study to another due to several factors such as the low number of patients ascertained to the study, selection bias of subjects, the STS markers used, different geographical regions, environmental factor, occupational exposures, and/or ethnicity of the study population [20, 25–27]. The frequencies of Y chromosome microdeletions differ from one study to another due to several factors such as the low number of patients ascertained to the study, selection bias of subjects, the STS markers used, different geographical regions, environmental factor, occupational exposures and/or ethnicity of the study populations. The potential mechanisms for this variation in Y chromosome microdeletion are both genetic and environmental. As no recombination occurs in the AZF region on the Y chromosome, the most likely source of variation could be attributed to the presence of highly repetitive DNA sequences which provide a source of variation between different studies according to STS markers that have been used in each study [28]. However, the usage of highly specific, non-polymorphic ones will provide more accuracy. Another factor that accounts for the variation in Y chromosome microdeletion is due to different origins of the studied population, their composition, and their geographical locations [29, 30]. The occupational risk of the study population such as pesticide and heavy metal exposure is also another determinant factor. Certain environmental factors for example smoking and using tobacco derivatives may predispose some men to have de-novo deletions [31]. Therefore, it is essential to study the environmental background of the patients to help in the proper investigation of the Y chromosome microdeletions [31].

However, the present microdeletion frequency is very close to a recent study among Iraqi infertile men where the Y chromosome microdeletions reached a frequency of 47.8% (43/90 infertile male patients) [32] and a previous one in the same population conducted in 2017 with a frequency of 65% (26/40) [33]. We found in the present study these microdeletions are more common in azoospermic than oligozoospermic and this in agreement with what was found in many reports [20, 22, 24].

In this study, we were able to identify deletions of the AZFa, AZFb, AZFc, and AZFd plus several combinations. Previous and recent studies reported a maximum of three region deletions [19, 24, 26, 32].

Deletions in the AZFa region were the most frequent microdeletions (71.4%) among the patient in our study, and it was significantly associated with Oligozoospermic phenotype. Though the deletion of AZFa is not common, a recent study in India has reported 3.2% AZFa deletions among its study population [34]. Another earlier study in the same country has reported a frequency of 17.2% [35]. In previous studies, AZFa region deletions were the least common type of deletions and in these cases, Azoospermia is frequently accompanied by germ cell aplasia and the presence of Sertoli cells in the seminiferous tubules [36].

The deletion of the AZFc region was significantly associated with the Azoospermia phenotype in our study with a frequency of 56.3%. Several studies have shown that AZFc deletion was invariably the most common [18, 19, 21, 22, 27]. The deletion of AZFc was variably associated with Sertoli-cell-only syndrome (SCOS), the developmental arrest of germ cells at the spermatid stage, and maturation arrest [37].

We were able in this study to add more information about the frequency of AZFd of 23.3% which is rarely analyzed in previous studies [38, 39]. Other studies showed conflicting results [20, 32, 40].

The most frequent combination was AZFbc, followed by AZFbcd, while AZFabc and AZFbd sharing the same frequency within the patients. Similar results were obtained from Iran for the high frequency of combination AZFbc followed by AZFabc [41]. Among Azoospermic participants, the AZFbc microdeletions (18.8%) were the most frequent followed by AZFbcd (12.5%) and AZFabc was the lowest frequent deletion with a frequency of 6.3%. AZFbd and AZFac deletion combination were not detected in the Azoospermic participants. These findings are in agreement with the previous study, where AZFbc and AZFabc deletions were found in non-obstructive azoospermic males [42]. Deletions in the AZFbc and AZFabc can cause chromosomal instability and can be responsible for chromosomal rearrangements or Y chromosome loss. Among Oligozoospermic the reported combinations were AZFbd and AZFac at a frequency of 7.1%. No AZFbc, AZFbcd, AZFabc deletion combination was detected in this study.

The Prolactin, LH, and FSH levels in AZF deleted males seem to vary in different studies. Serum levels of PRL, LH, and FSH were compared between (Azoospermic and Oligozoospermic—with or without microdeletion).

Generally, hormonal analysis in our study has shown that prolactin was slightly higher than the normal level in azoospermia participants with microdeletion. The effect of hyperprolactinemia is that it suppresses both FSH and LH and reduces spermatogenesis [43]. We recorded 56.3% of Azoospermic have higher prolactin levels compared to 14.3% in the Oligozoospermic and a very close frequency to this of the Azoospermic (64%) was revealed to have the AZF microdeletion. A weak negative correlation was detected between prolactin higher level and Azoospermic patients r = 0.434, *p* = 0.016. Moreover, a very weak correlation was detected between prolactin and the following AZF regions (AZFa r = 0.665 and 0.602, *p* = 0.000 and 0.0004, AZFb r = 0.636 and 0.409, *p* = 0.000 and 0.025, and AZFd r = 0.398 and 0.442, *p* = 0.029 and 0.015). The deletion of AZF is characterized by the inability to produce sperm, resulting in no sperm in the semen [44]. Among the participants without microdeletion, an equal frequency of 33.3% in both Azoospermic and Oligozoospermic have a higher prolactin level. However, this correlation should be carefully discussed given the small sample size.

LH level was higher than the normal range in all participants with and without microdeletions. In general, Oligozoospermic without microdeletion was revealed the highest level of serum LH. Moreover, the LH level was higher in 53.8% of Azoospermic compared to 50% in Oligozoospermic among participants with microdeletion. The previous study has shown that levels of FSH and LH in infertile patients with deletions were significantly higher than those in participants without microdeletions [42].

FSH serum levels were higher than the normal range in all participants with and without microdeletions except for the Oligozoospermic without microdeletion. We also observed a much higher FSH level in the group with YCDs compared with the other groups. FSH level was higher in 50% of Azoospermic compared to 42.9% in the Oligozoospermic among participants with microdeletion. In a previous study, the profound testicular failure reported among the study participants is reflected by their high mean FSH level [41]. Moreover, appropriate serum FSH levels govern the appropriate induction and maintenance of sperm production. It has been shown that azoospermic patients with high FSH levels = 20 IU/L have lower chances of having live-born children with the ICSI method [45, 46]. Y chromosome microdeletions cause impaired spermatogenesis which has been known to cause high FSH levels. The variation in male hormone physiology has been shown in earlier studies to be affected by microdeletions of the Y chromosome according to levels of FSH, LH, and T [47, 48]. One of the good examples is the microdeletion of AZFb + c + d which was found to cause a high level of the high level of FSH [49]. The nature of this relationship between Y chromosome microdeletion and reproductive hormone levels needs advanced investigations.

In the present study, we didn’t observe a significant correlation between the mean values of the three hormones with the presence or absence of the AZF microdeletion. This is in agreement with the results of previous studies [42, 50]. Variations in male hormone physiology as indicated by the levels of FSH, LH and T plus to modification of gonadal morphology have been revealed in previous studies in patients having microdeletions of the Y chromosome [47, 48]. Advance research is needed to find out the type of correlation between Y microdeletions in infertile patients and variations of reproductive hormone levels with careful consideration to the selected control groups. They should be consisted of patients with the same clinical features, as patients under study, except for the microdeletion. It might be possible then, to exclude that the microdeletions per se would affect spermatogenesis and hormonal levels in a different fashion than other known andrological conditions.

## Conclusion

We concluded in this study that the incidences of microdeletions of the Y chromosome confined to AZF a, b, c and d regions is 58.8% in infertile subjects with 31.4% were Azoospermic and 27.5% were Oligozoospermic. This might provide a piece of evidence that these specified regions of the Y chromosome are essential for controlling spermatogenesis. The significance of the present study was remarked by the most frequent percentage of deletions detected in the AZFc region (56.3%) among Azoospermic participants and that was significantly associated with Azoospermia phenotype. These results suggest that microdeletions of the Y chromosome may be associated with testicular sperm production. However, further studies on environmental factors including ethnic differences are needed as infertility is considered a multifactorial problem. Genetic counseling and advice for appropriate methods that could be used in assisted reproduction are required to avoid pointless treatments and vertical transmission of Y chromosome abnormalities to the offspring. Practical application will be detailed for Sudanese IVF centers to make use of findings from this study in the routine clinical workup of both oligozoospermic and azoospermic patients.

## Data Availability

The datasets used and/or analyzed during the current study are available from the corresponding author on reasonable request.

## Abbreviations

AZF: Azoospermia factors
EDTA: Ethylenediaminetetraacetic acid, a chelating agent
ELISA: Enzyme-linked immunosorbent assay
FSH: Follicle Stimulating hormone
LH: Luteinizing hormone
m-PCR: Multiplex polymerase chain reaction
PRL: Prolactin hormone
SCOS: Sertoli-cell-only syndrome
SRY: Sex-determining region Y
STS: Sequence-tagged sites
TBE: Tris–Borate-EDTA
WHO: World Health Organization
